# Analysis of the Memoirs of the Medical Society of London

**Published:** 1799-09

**Authors:** 


### *Analysis of the Memoirs of the Medical Society of London.*

[Continued from our last Number, pp. 29---35.]

Art. XX. contains "*Caſes of Cynanche Trachealis, ſucceſsfully treated, with Obſervations on that diſeaſe*," By H. Field, Apothecary, Sec. M. S.

The treatment of theſe caſes is by veneſection, leeches, bliſters to the ſternum, antimonial emetics, laxatives when neceſſary, and an embrocation to the throat, by means of linen cloths, conſtantly wetted with

℞ Aq. ammon. acet. unc. ij.—Spir. æther. vitr. comp. unc. j. M.

After the account of four caſes, and the ſucceſsful treatment, Mr. Field adds,

"Previouſly to entering upon thoſe practical obſervations which are deſigned to accompany theſe caſes, it will not, I truſt, be deemed totally unintereſting, to premiſe a few remarks on the different kinds of diſeaſe which have been known under the general name of croup, and alſo on the nature of the malady as to contagion.

"It has been ſtated by authors, that there are two kinds of this diſeaſe, the one ſpaſmodic, and the other inflammatory. Of the propriety of this diſtinction I am at preſent well ſatisfied, and am only concerned, that two diſeaſes, ſo extremely different in their cauſes, and conſequently in their mode of treatment, ſhould be confounded under one and the ſame title; a circumſtance which has an evident tendency to miſlead the practitioner, and which has undoubtedly been the reaſon why we find ſuch very oppoſite modes

---

modes of cure recommended by different writers, and each with a confidence derived from fome degree of experience.

" That the fymptoms of the two difeafes bear confiderable refemblance, will readily be allowed; neverthelefs, there are evidences of difference, I think, fufficiently ftrong to enable an attentive perfon to difcriminate them. Thefe marks of diftinction I fhall endeavour now to defcribe, and to give them the greater effect, fhall contraft the one with the other.

" The fpafmodic croup always attacks fuddenly, and ufually in the night. The attack of the inflammatory croup is fometimes equally fudden, but more generally gradual, being preceded a few days by flight feverifh fymptoms, and a teafing, fhort cough, not however, fufficiently important to create the fmalleft uneafinefs in the friends of the patient. The fpafmodic croup often intermits; and in thefe intervals, both the refpiration and the cough, if any exifts, are free from its ufual characteriftic found: the inflammatory, on the contrary, when once completely formed, never intermits fo as entirely to lofe its diftinguifhing mark, particularly in coughing; add to which, the heat, frequency of pulfe, and other fymptoms of *pyrexia*, are found in the latter in much greater degree than in the former. Dr. RUSH * has mentioned feveral other marks of difference, but as they apply chiefly to the effect of remedies, and to the later ftages of the difeafe, it is not judged neceffary to infift upon them here, it being in the firft attack of this malady, that a due difcrimination becomes fo extremely important; that being the time in which the application of powerful and decifive remedies is moft conducive to the relief of the afflicted, a delay of a few hours being frequently the caufe of irreparable injury.

" Every author that I am at prefent acquainted with, has denied this difeafe to be of an infectious nature. In a former † paper on this fubject, I have taken the liberty to fuggeft my doubts as to this opinion being well founded, for which I have there affigned reafons; fince that time, my particular attention has been given to that point, and I am forry to add, that increafed experience has tended to confirm me more ftrongly in the opinion, that the true *cynanche trachealis* is a contagious difeafe. I have fince met with repeated inftances of its occurring in the fame family, and that after fuch an interval as we moft ufually find contagious difeafes to require in order to produce their morbid effects, namely, from fix to ten days: whether the above opinion be well or ill founded, I would ftrongly recommend to practitioners to avoid the danger of communication, by requefting that every child may be removed, if poffible, from the fame houfe; or, at all events, be prevented entering or coming near to the fickchamber.

" It has been faid that this difeafe has occafionally been met with in adults; when this has been the cafe, I am very much difpofed to think, that it was not the

* Vide " Medical Inquiries," by B. Rush, M. D. vol. i. p. 141.

† Vide " Memoirs of the Medical Society of London," vol. iv. p. 151.

the inflammatory, but the ſpaſmodic croup; in confirmation of which opinion, I have never heard of its having proved fatal to them.

"The firſt and moſt important curative indication in the treatment of the true or inflammatory croup (for to this our preſent obſervations will be confined) is, to diminiſh the quantity of blood. In the paper on this diſeaſe, which the Society have done me the honour to publiſh in their Memoirs, I gave a caution againſt the uſe of the lancet, from an apprehenſion that the early debility, which had been obſerved to come on, would render general bleeding an unſafe and improper practice, and that our evacuation of blood ſhould be only topical, by means of leeches; which, however, was adviſed to be freely and vigorouſly purſued. Since that time I have had opportunities of obſerving, that the lancet may not only be ſafely, but even advantageouſly employed, and that it ſhould never therefore be omitted, when medical advice is required in the earlier ſtages of the diſeaſe, from two to four or five ounces of blood being taken away, according to the age and ſtrength of the patient; much caution is nevertheleſs requiſite in repeating this operation. If any abatement of ſymptoms takes place after the firſt bleeding, which frequently happens, I ſhould certainly think it unneceſſary to repeat that evacuation; but if an evident exacerbation ſhould afterwards come on, it will be generally proper to do ſo: in this caſe a topical diſcharge, by means of leeches, appears to me much to be preferred to a general one. Allow me here to give a caution relative to the prognoſis in this diſeaſe. The means now recommended in the early ſtage of it, being frequently followed by a conſiderable and very flattering appearance of recovery, the practitioner may be ſo far deceived as to be encouraged himſelf, and, in conſequence, to encourage the friends of the patient, with great expectation of a favourable iſſue; but in this he cannot be too much on his guard, nor ſhould he conſider the danger to be paſt, until three or four days have elapſed without a return of ſymptoms, by which time the patient will have made conſiderable progreſs towards recovery.

"Our next ſubject will be an inquiry into the uſe of bliſtering in the cure of this diſeaſe. I have, on a former occaſion *, taken notice of Dr. HOME's objection to the early application of bliſters to the affected parts, as liable to do injury by their immediate ſtimulus. I am well ſatisfied, from later obſervations, that this objection is well founded, although ſufficient attention does not ſeem in general to have been given to it; and whoever conſiders the extreme vicinity of the diſeaſed part the external ſurface of the throat, muſt ſurely coincide with me in opinion, that the application of a bliſter immediately to the part muſt act as a local ſtimulus, and therefore muſt increaſe, rather than diminiſh inflammation. Veſicatories ſhould, for theſe reaſons, be either entirely omitted, or elſe applied only to diſtant parts. Whether they will in the latter caſe be of any ſervice, I am at preſent unable to aſcertain. Bliſters were applied in only two

* Vide "Med. Soc. Memoirs," vol. iv. p. 159.

two of the prefent cafes, and in thofe there is not the fmalleft reafon to fuppofe, that they contributed in any degree to the cure; in the laft of the two, the blifter fcarcely took any fenfible effect on the fkin.

"The fituation of the trachea with refpect to the external integuments, which I have above alluded to, fuggefted to me an idea that refrigerating, and alfo fedative remedies, might be ufed externally with advantage. In the third and fourth cafes now recited, I made trial of an embrocation with that intention; how far the fuccefs of thofe cafes is to be attributed to this remedy, it is impoffible to fay; it is fufficient, however, to enable me to recommend this and fimilar applications to further trial, and alfo emollient and fedative cataplafms and fomentations. The occafional ufe of emetics, fo as to produce their full effect, and their conftant ufe fo as to excite naufea, as far as has hitherto appeared, feems to be attended with good confequences. The body fhould be kept at all times in a foluble ftate, but any confiderable evacuation by ftool is better avoided, its immediate tendency being to debilitate, without apparent advantage, in relieving the patient. The warm bath, either partial or general, may be employed with probability of benefit."

ART. XXVII. Prefents us with fome very interefting and new "*Obfervations on human inteftinal Worms; being an attempt to their arrangement into Claffes, Genera, and Species.*" By R. HOOPER, M. D.

As this article is illuftrated by beautiful plates which we cannot command, we muft content ourfelves with prefenting our readers with the refult of Dr. Hooper's claffification, referring to the Memoirs themfelves for the fynonyms, anatomy, &c.

Worms found in the human ftomach and inteftines he divides into two claffes, viz. into,

I. Thofe worms, which are generated and nourifhed in the human inteftinal canal, and which there propagate their fpecies; and,

II. Thofe infects or worms, that accidentally enter the human primæ viæ *ab extra*; fuch are, feveral fpecies of fcarabæi, the lumbricus terreftris, the fafciola, the gordius inteftinalis, and others. This fecond clafs belongs to the province of natural hiftory, and is therefore omitted by Dr. Hooper.

The firft clafs is divided into *two orders,* viz.

Order I. ROUND WORMS.

Genus 1. *Afcaris.*

Species 1. Afcaris *lumbricoides.* The long round worm.

2. —— *vermicularis.* The thread-worm.

Genus

Genus 2. *Trichuris.*

Species 1. Trichuris *vulgaris.* The long thread-worm.

Order II. Flat Worms.

Genus 1, *Tape worms.*

Species 1. *Tænia osculis marginalibus.* The long tape-worm.

2. ———— *superficialibus.* The broad tape-worm.

This volume contains a collection of most interesting papers, and histories of particular cases of disease; but whenever we make any extracts from the few select works which we profess to analyze, we endeavour to confine ourselves as much as possible, to improvements in the *practice* of medicine and pharmacy.

